# A role for cathepsin Z in neuroinflammation provides mechanistic support for an epigenetic risk factor in multiple sclerosis

**DOI:** 10.1186/s12974-017-0874-x

**Published:** 2017-05-10

**Authors:** Euan R. O. Allan, Rhiannon I. Campden, Benjamin W. Ewanchuk, Pankaj Tailor, Dale R. Balce, Neil T. McKenna, Catherine J. Greene, Amy L. Warren, Thomas Reinheckel, Robin M. Yates

**Affiliations:** 1 Snyder Institute for Chronic Disease, University of Calgary, Calgary, AB T2N 4 N1 Canada; 2 Department of Biochemistry and Molecular Biology, Faculty of Medicine, University of Calgary, 3330 Hospital Drive NW, HRIC 4AA10, Calgary, AB T2N 4 N1 Canada; 3 Department of Veterinary Clinical and Diagnostic Services, Faculty of Veterinary Medicine, University of Calgary, Calgary, AB T2N 4 N1 Canada; 4 Institute of Molecular Medicine and Cell Research, Faculty of Medicine, Albert-Ludwigs-University, D-79104 Freiburg, Germany; 5 BIOSS Centre for Biological Signalling Studies, Albert-Ludwigs-University, D-79104 Freiburg, Germany

## Abstract

**Background:**

Hypomethylation of the cathepsin Z locus has been proposed as an epigenetic risk factor for multiple sclerosis (MS). Cathepsin Z is a unique lysosomal cysteine cathepsin expressed primarily by antigen presenting cells. While cathepsin Z expression has been associated with neuroinflammatory disorders, a role for cathepsin Z in mediating neuroinflammation has not been previously established.

**Methods:**

Experimental autoimmune encephalomyelitis (EAE) was induced in both wildtype mice and mice deficient in cathepsin Z. The effects of cathepsin Z-deficiency on the processing and presentation of the autoantigen myelin oligodendrocyte glycoprotein, and on the production of IL-1β and IL-18 were determined in vitro from cells derived from wildtype and cathepsin Z-deficient mice. The effects of cathepsin Z-deficiency on CD4+ T cell activation, migration, and infiltration to the CNS were determined in vivo. Statistical analyses of parametric data were performed by one-way ANOVA followed by Tukey post-hoc tests, or by an unpaired Student’s *t* test. EAE clinical scoring was analyzed using the Mann–Whitney *U* test.

**Results:**

We showed that mice deficient in cathepsin Z have reduced neuroinflammation and dramatically lowered circulating levels of IL-1β during EAE. Deficiency in cathepsin Z did not impact either the processing or the presentation of MOG, or MOG- specific CD4+ T cell activation and trafficking. Consistently, we found that cathepsin Z-deficiency reduced the efficiency of antigen presenting cells to secrete IL-1β, which in turn reduced the ability of mice to generate Th17 responses—critical steps in the pathogenesis of EAE and MS.

**Conclusion:**

Together, these data support a novel role for cathepsin Z in the propagation of IL-1β-driven neuroinflammation.

**Electronic supplementary material:**

The online version of this article (doi:10.1186/s12974-017-0874-x) contains supplementary material, which is available to authorized users.

## Introduction

Enigmatic to the pathogenesis of multiple sclerosis (MS) are the mechanisms that link known risk factors to the incidence and development of this immune-driven demyelinating disease. Since the incidence of MS is influenced by environmental factors and gender, but has a low concordance rate in monozygotic twins and underwhelming odds ratios for individual SNPs, epigenetic changes are likely to play a major role in determining an individual’s susceptibility to MS [1]. In 2014, Huynh et al. compared epigenomic differences between pathology-free regions of healthy and MS-affected brains in an attempt to identify potential epigenetic risk factors for MS [2]. One of the most significant findings was that the cathepsin Z (CTSZ) locus was hypomethylated in pathology-free regions of MS patients, which resulted in increased expression of cathepsin Z within this neural tissue [2]. While the underlying mechanism that results in hypomethylation at this particular locus is unknown, the authors proposed that the epigenetically-driven expression of cathepsin Z in neural tissue may increase an individual’s susceptibility to MS.

Cathepsin Z (also known as cathepsin X) was identified in silico by its similarity to the family of cysteine-type lysosomal proteases, through mining the Expressed Sequence Tags database from human brain tissue [3, 4]. Cathepsin Z is a unique member of this 11 member-protease family, as it is the only enzyme with strict carboxypeptidase activity, it has a remarkably short pro-domain that contains a RGD integrin binding domain, and the CTSZ gene is chromosomally separated from the other lysosomal cysteine cathepsin genes [5–8]. In the context of neoplasia, there is recent in vivo evidence for a tumor-promoting role for the carboxypeptidase as well as for the RGD function of cathepsin Z [9]. However, to date, the specific functions of cathepsin Z within the central nervous system (CNS) remain obscure.

Whilst there was mounting evidence to support the association of cathepsin Z expression with neuroinflammation [10–13], whether cathepsin Z had a specific pathogenic role in neuroinflammatory disorders was erstwhile unknown. Here, we present experimental evidence to support a non-redundant role for cathepsin Z in neuroinflammation in mice. In a model of multiple sclerosis—experimental autoimmune encephalomyelitis (EAE)—mice deficient in cathepsin Z consistently developed lower levels of neuroinflammation and displayed disproportionally lower levels of circulating IL-1β. The ability to generate IL-1β in response to NLRP3-stimulus by macrophages and dendritic cells derived from cathepsin Z-deficient mice was compromised, as was the ability of cathepsin Z-deficient mice to generate Th17 responses. Collectively, these data indicate that cathepsin Z promotes the IL-1β–Th17 axis leading to more severe neuroinflammation during EAE in mice and may suggest a role for cathepsin Z in the development of MS, as proposed by Huynh et al. [2].

## Materials and methods

### Mice and cells

C57BL/6 (wildtype [WT]) and C57BL/6-Tg(Tcra2D2,Tcrb2D2)1Kuch/J (2D2) mice were purchased from the Jackson Laboratory (Bar Harbor, ME, USA). 2D2 mice express a transgenic CD4+ T cell receptor (Vβ11 TCR/Vα3.2 TCR) that is specific for the immunodominant MOG^35–55^ peptide in the context of I-A^b^ [14]. Cathepsin Z-deficient mice (Cat Z^−/−^) were generated as previously described [15]. In brief, a segment of the murine cathepsin Z exon 2, containing a portion of the active site along part of intron 3, was substituted with a ribosomal entry sequence [15]. Cathepsin Z-deficient mice were also crossed with 2D2 mice, generating mice with cathepsin Z-deficient 2D2 CD4+ T cells. All mice used were fully backcrossed to the C57BL/6 background, and bred and housed under identical animal husbandry conditions. All animal research was performed in accordance with the Canadian Council for Animal Care, and protocols were approved by the University of Calgary Animal Care and Use committee. All mice were age and sex matched within experiments, and used between the ages of 8 and 12 weeks. All mice subject to in vivo studies were 8–10 weeks of age [16, 17]. Bone marrow-derived macrophages (BMMØs) were derived from bone marrow using L929-conditioned media and activated for 18 h with recombinant IFNγ (rIFNγ) (100 U/ml, Pepro Tech), as previously described [18, 19]. Bone marrow-derived dendritic cells (BMDCs) were derived using media conditioned with the supernatant of Ag8.653 myeloma cells transfected with GM-CSF cDNA, as previously described [16, 20–22]. Peritoneal macrophages (pMØs) were isolated from the peritonea of mice by injection of 8 mL of sterile PBS using a 23 g needle, followed by removal of 5 ml of PBS/peritoneal fluid after brief abdominal massage [23]. The murine microglia-like cell line BV2 (C8-B4 [ATCC® CRL-2540™]) was grown in RPMI supplemented with 5% FBS. The murine albino neuroblastoma cell line Neuro-2a (N2A) (ATCC® CCL-131™) was grown in DMEM/F-12 supplemented with 5% FBS. The murine dendritic cell-like DC2.4 (CVCL_J409) was grown in RPMI supplemented with 5% FBS, 10 mM β-mercaptoethanol (2-ME), 20 mM L-glutamine and 100 mM HEPES [24]. All cells were cultured at 37 °C with 5-7% CO_2_.

### Flow cytometry

Flow cytometry was performed using a FACSCalibur flow cytometer (BD Biosciences, Franklin Lakes, NJ, USA) and analyzed with FLOWJO software v8.6 (Tree Star, Ashland, OR, USA) [16]. Leukocyte populations, with a minimum of 2.5 × 10^4^ counts, were selected using forward scatter/side scatter (FSC/SSC). All antibodies were purchased from BD Biosciences [16].

### Assessment of MOG antigen processing and presentation

MOG antigen presentation efficiency was assessed by measuring the relative level of activation of CD4+ T cells derived from the TCR transgenic mouse model 2D2 following co-culture with MOG-pulsed antigen presenting cells (APCs), as previously described [14, 16, 25–27]. In brief, WT and Cat Z^−/−^ BMMØs and BMDCs were exposed for 6 h to medium containing the immunodominant I-A^b^ peptide epitope MOG^35–55^ (0, 1, 10, 25 μg/ml), synthesized by the University of Calgary Peptide Services (AB, Canada), or the full extracellular domain of MOG (MOG^1–125^; 0, 1, 10, 25 μg/ml), prepared as previously described [16]. APCs were then washed with T cell media (RPMI supplemented with 10% FBS and 10 mM 2-ME), and naïve 2D2 or Cat Z^−/−^ 2D2 splenocytes were added to the APCs and incubated for 16 h [16]. The presentation efficiency of MOG^35–55^ by the WT and Cat Z^−/−^ APCs, to the 2D2 or the Cat Z^−/−^ 2D2 CD4+ T cells, was determined cytometrically using the surface expression of the early activation marker, CD69.

### CD4+ T cell trafficking

CD4+ T cell trafficking was assessed as previously described [28]. In brief, WT and Cat Z^−/−^ CD4+ T cells were isolated from spleens of WT and Cat Z^−/−^ mice using the EasySep Mouse CD4+ T Cell Enrichment Kit (StemCell Technologies), according to the manufacturer’s instructions. 3 × 10^5^ CD4 + T cells were transferred to the upper filter of a 5 μm Transwell support plate (Corning) pre-coated overnight with 3 μg/ml ICAM-Fc (R&D Systems). T cells were allowed to settle for 30 minutes before the upper filter was exposed to the bottom chamber containing vehicle (PBS) or 1 μg/ml CXCL9 (R&D Systems), followed by an incubation period of 1 h at 37 °C. The absolute numbers of cells that migrated through the Transwell filter were enumerated with a hemocytometer.

### Induction of EAE and adoptive transfer of 2D2 CD4+ T cells

EAE was induced in WT and Cat Z^−/−^ mice using standard protocols, as previously described [16, 17]. In brief, 8- to 10-week-old female WT and Cat Z^−/−^ mice were anesthetized using intraperitoneal (i.p.) injection of ketamine-xylazine. Following anesthetization, each mouse was injected sub cutaneously (s.c.), in both flanks, with a 100 μL emulsion of 50 μg MOG^35–55^ in complete Freund’s adjuvant (0.5 mg/ml *M. butyricum* in paraffin oil) (CFA; BD). Additionally, each mouse received an i.p. injection of pertussis toxin (PT) (300 ng) on days 0 and 2, and the clinical score and weight was recorded daily for the duration of the experiment. The following clinical scoring system was used: score 0 - asymptomatic; 0.5 - tail weakness; 1 - limp tail; 1.5 - hind limb limping; 2 - hind limb weakness; 2.5 - partial hind limb paralysis; 3 - complete hind limb paralysis; 3.5 – hind limb paralysis with forelimb weakness; 4 – forelimb paralysis; 4.5/5 - complete morbidity/death [16, 17]. Mice that received saline/CFA/PT did not develop clinical signs of EAE (data not shown). Additional cohorts of mice were sacrificed at day 15 for CNS leukocyte analysis and cardiac puncture for Luminex analysis of peripheral cytokines [16]. To investigate the ability of WT and Cat Z^−/−^ mice to generate Th17, Th1 and Treg CD4+ T cell responses in the absence of MOG, anesthetized mice were injected s.c., in both flanks, with a 100 μL saline/CFA emulsion. These mice were sacrificed 6 days after injection, the inguinal lymph node cells (LNC) were removed and CD4+ T cells expressing IL-17, IFNγ, and FoxP3 were counted by flow cytometry. To examine CD4+ T cell migration to and activation within the CNS, 2D2 and Cat Z^−/−^ 2D2 CD4+ T cells were activated, expanded, and adoptively transferred into WT or Cat Z^−/−^ mice, as previously described [29]. Briefly, 2D2 and Cat Z^−/−^ 2D2 splenocytes were harvested and cultured in T cell medium (RPMI supplemented with 10% FBS and 10 mM 2-ME) containing 20 μg/ml MOG^35–55^ and 0.5 ng/ml IL-12 (R&D Systems) for 48 h. The expanded 2D2 or Cat Z^−/−^ 2D2 CD4+ T cells were injected into the peritoneal cavities of WT or Cat Z^−/−^ mice (5 × 10^6^ total cells/mouse suspended in 100 μL PBS) [16]. Adoptively transferred CD4+ T cells were isolated 6 days later using a discontinuous Percoll gradient, immunostained for CD4+, Vα3.2+ (2D2 TCR) and CD25, and analyzed by flow cytometry.

### Spinal cord leukocyte profiling

Infiltrating and resident leukocytes of the spinal cord were isolated 15 days post EAE induction using a discontinuous Percoll gradient, as previously described [16, 30]. These cells were immunostained in the following combinations: macrophages (CD11b+/CD45+ high), B cells (B220+/CD45+), CD8+ T cells (CD8+/CD3+), CD4+ T cells (CD4+/CD3+) and Th17 cells (IL-17+/CD4+/CD3+), and analyzed by flow cytometry.

### Histology

The thoracolumbar spinal cord of each WT and Cat Z^−/−^ mouse, sacrificed at day 15 after EAE induction, was removed in toto and fixed in 10% neutral buffered formalin. Transverse sections of the lumbosacral spinal cord from the lumbar intumescence were paraffin embedded, sectioned at 3 μm and stained with hematoxylin and eosin (HE) and Luxol fast blue (LFB) (Prairie Diagnostic Services, Saskatoon, SK, Canada).

### Fluorometric assessment of phagolysosomal proteolysis

Proteolytic efficiencies of phagolysosomes in live phagocytes were measured, as previously described [18, 19]. In brief, WT and Cat Z^−/−^ BMDC and BMMØs were allowed to phagocytose 3 μm silica beads that were covalently coupled to human IgG (Sigma) and to the fluorogenic protease substrate DQ Green BODIPY albumin (DQ-albumin; Invitrogen) bearing the reference fluor (Alexa Fluor 594 succinimidyl ester; Invitrogen). The proteolytic efficiencies of the resulting phagolysosomes were determined by measuring the green fluorescence liberated from hydrolysis of the particle-bound DQ-albumin, relative to that of the reference fluor, over a one-hour period. Measurements were performed at 37 °C in microplate format using an Envision multilabel plate reader (PerkinElmer Life Sciences).

### qPCR

Quantitative PCR (qPCR) was used to quantify key mRNA transcripts in spinal cord tissue over the course of EAE, as well as in WT and Cat Z^−/−^ APCs in response to LPS [16]. In brief, WT lumbar spinal cords, spleens, neurons or APCs (including pMØs, DC2.4 s, BV2s, BMMØs and BMDCs treated with LPS for 3 h) were snap-frozen in liquid nitrogen, total RNA was extracted using the RNeasy lipid tissue mini kit (Qiagen), and cDNA was synthesized using iScript Reverse Transcriptase Supermix for RT-qPCR (BioRad). qPCR was performed as previously described [19]. All primers were prepared at 300 nM, had a single melt curve, had efficiencies between 90–100%, and were designed or verified using Primer 3 (National Center for Biotechnology Information). 18S (F: 5′- AGTCGGCATCGTTTATGGTC-3′; R: 5′-CGCGGTTCTATTTTGTTGGT-3′) was used as an internal control, and did not vary across treatments; IL-1β (F, 5′-CAACCAACAAGTGATATTCTCCATG-3′; R, 5′-GATCCACACTCTCCAGCTGCA-3′) and Cat Z (F, 5′- CCTGTCCGGGAGGGAGAA −3′; R, 5′- TGGTTGATAACGGCCTGGTC −3′) [31] were amplified using the following PCR conditions (in a BioRad iQ5 thermocycler): 95 °C for 5 min; 40 cycles of 95 °C for 30s and 55 °C (58 °C for 18S and IL-1β) for 30s. All mRNA levels were presented relative to 18S and the WT control samples.

### IL-1β and IL-18 generation by APCs

APC generation of IL-1β and IL-18 in response to activation of the NLRP3 inflammasome in vitro were quantified by Mouse IL-1β ELISA and Mouse IL-18 Platinum ELISA (eBioscience) [32]. In brief, WT and Cat Z^−/−^ BMMØs, and BMDCs were pre-incubated with 200 ng/ml of ultra-pure LPS from *Salmonella minnesota* R595 (List biological laboratories) for 3 h. Following a single wash with warm PBS, BMDCs were incubated with adenosine triphosphate (ATP 5 mM) for 1 h (Sigma-Aldrich); and BMMØs with monosodium urate crystals (MSU, 300 ng/ml prepared as in [33]) for 6 h in appropriate media. Supernatants were collected and protein concentrations were measured by ELISA according to the manufacturer’s instructions.

### Statistical analysis

Statistical analyses were performed by one-way ANOVA with a Tukey post hoc test, or an unpaired Student’s *t* test, as specified (*p* < 0.05). If a Bartlett’s test for equal variance failed, then the data underwent a natural log transformation before reanalysis. Analyses of EAE clinical scoring were performed using the non-parametric Mann–Whitney *U* test [16]. Analyses were completed using GraphPad Prism software (La Jolla, CA, USA).

## Results

### Cathepsin Z does not significantly impact proteolytic efficiencies within phagolysosomes of macrophages

**Fig. 1:**
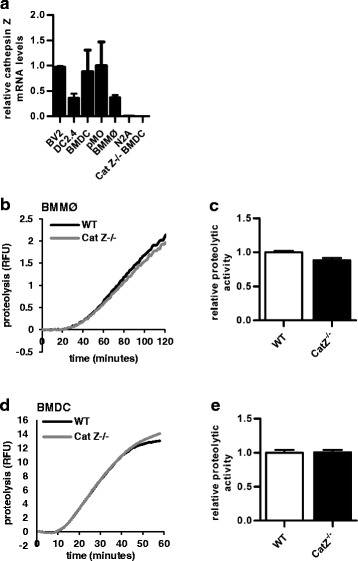
Cathepsin Z is highly expressed in APCs but does not significantly contribute to phagolysosomal proteolysis. **a** Cathepsin Z mRNA levels in BV2 (C57BL/6 brain microglia cell line), DC2.4 (dendritic cell line), BMDC (bone marrow derived dendritic cells), pMØs (peritoneal macrophages), BMMØ (bone marrow derived macrophages), N2A (murine albino neuroblastoma cell line) and Cat Z^−/−^ BMDCs (*n* = 3). **b**-**e** The total proteolytic activity (rate of substrate-liberated fluorescence from the particle-bound fluorogenic substrate DQ-albumin) following phagocytosis of fluorometric experimental particles in WT and Cat Z^−/−^ (**b**-**c**) BMMØ (*n* = 9) and (**d**-**e**) BMDC (*n* = 5). **b**, **d** Representative real-time traces of phagosomal proteolysis. **c**, **e** Averaged rates of proteolysis (determined by calculation of the slope of the linear portion of the real-time trace [as described by y = mx + c, where y = relative fluorescence, m = slope, and x = time] were calculated between (**c**) 20 min and 60 min or (**e**) 20 min and 40 min after particle internalization. Data presented as mean+/− SEM; (**c**, **e**) no significant differences (unpaired Student’s *t*-test, *p* > 0.05) from the WT control were observed

The lysosomal cysteine protease, cathepsin Z, has been reported to be highly expressed in antigen presenting cells (APCs) [12, 13, 34]. This was confirmed in peritoneal and bone marrow-derived macrophages (pMØ, BMMØ), immortalized and bone marrow-derived dendritic cells (DC2.4, BMDC) and immortalized microglia (BV2) (Fig. 1a). Although the functions of cathepsin Z are ill-defined, it is well accepted that the primary function of many lysosomal cysteine cathepsins—such as cathepsin B, L and S—is to facilitate protein turnover by hydrolyzing self and foreign proteins in the cellular endolysosomal network. This system is particularly well developed in phagocytic APCs such as macrophages and dendritic cells, where proteolytic cleavage by these enzymes within phagosomes and endosomes is also necessary for the processing of T cell antigens [35–37]. In order to determine the relative contribution of cathepsin Z to endolysosomal proteolysis, the hydrolysis of phagocytosed protein was measured in WT and Cat Z^−/−^ BMMØs and BMDCs. Unlike specific deficiencies in other lysosomal cysteine cathepsins (such as cathepsin S), BMMØs and BMDCs derived from mice deficient in cathepsin Z displayed comparable efficiencies of phagosomal proteolysis (Fig. 1b–e).

### Mice deficient in cathepsin Z show attenuated neuroinflammation during EAE

**Fig. 2:**
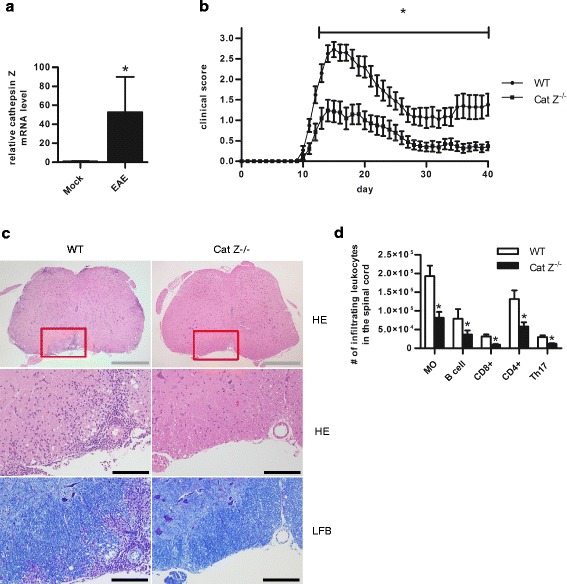
Cathepsin Z expression is increased in the CNS during EAE, and mice deficient in cathepsin Z exhibit attenuated signs of neuroinflammation and demyelination during EAE. **a** Cathepsin Z mRNA levels in the spinal cord tissue of WT mice 15 days after induction of EAE or mock (*n* = 6). **b** Clinical disease course of WT and Cat Z^−/−^ mice after active induction of EAE (*n* = 20–21). **c** Representative micrographs of transverse sections of lumbar spinal cord from WT and Cat Z^−/−^ mice at 15 days after induction of EAE. Sections are stained with hematoxylin and eosin (HE) for inflammation, or Luxol fast blue (LFB) for demyelination. *Grey* and *black* scale bars indicate 500 and 100 μm respectively. **d** The total number of infiltrating macrophages (MO), B cells, CD8+ T cells (CD8+), CD4+ T cells (CD4+), and Th17 cells (IL-17+/CD4+) isolated from lumbar spinal cord tissue 15 days post EAE induction as analyzed by flow cytometry (*n* = 8–12). Data presented as mean +/− SEM; significant differences (unpaired Student’s *t* test; clinical data, Mann–Whitney *U* test; *p* < 0.05) from the WT control are denoted by *asterisks*

In concordance with a previous microarray study [38], cathepsin Z mRNA was found to be significantly upregulated in neural tissue during murine EAE following induction with myelin oligodendrocyte glycoprotein peptide (MOG^35–55^) using qPCR (Fig. 2a). To determine whether cathepsin Z plays an active role in the pathogenesis of EAE or is merely an indicator of neuroinflammation, several parameters of neuroinflammation were compared between C57BL/6 (WT) and congenic cathepsin Z-deficient (Cat Z^−/−^) mice following the induction of EAE. Consistent with a potential role for cathepsin Z in neuroinflammation, Cat Z^−/−^ mice displayed significantly attenuated progression of the clinical signs of EAE compared to that of WT mice (Fig. 2b, Additional file 1: Figure S1). At 15 days post induction (EAE peak), spinal cord tissue in Cat Z^−/−^ mice showed reduced demyelination and neuroinflammation by histopathology, as well as diminished infiltration of macrophages and lymphocytes, as determined by flow cytometry (Fig. 2c, d).

### Cathepsin Z deficiency does not impact either the processing and presentation of the autoantigen MOG or CD4+ T cell activation and trafficking

**Fig. 3:**
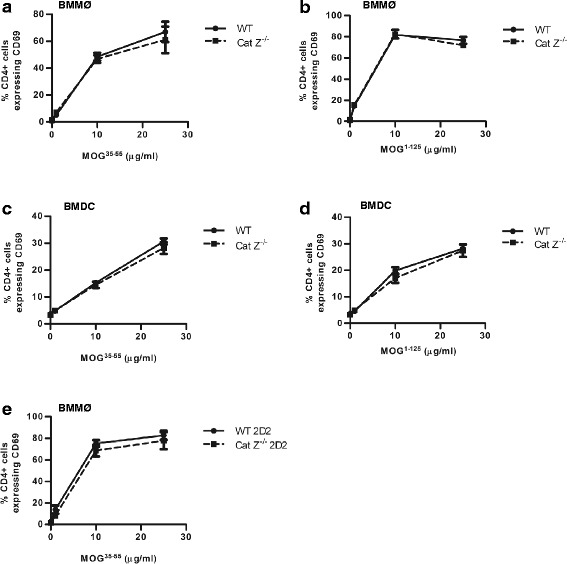
Cathepsin Z-deficiency does not affect the efficiency of MOG antigen processing or presentation in BMMØ and BMDC, or the efficiency of MOG-specific CD4+ T cell activation. **a-e** WT and Cat Z^−/−^ (**a**, **b**, **e**) BMMØ and (**c**-**d**) BMDC were incubated with (**a**, **c**, **e**) MOG^35–55^ peptide (0, 1, 10, 25 μg/ml), or (**b**, **d**) recombinant MOG^1–125^ (0, 1, 10, 25 μg/ml) for 6 h before co-incubation with (**a**-**d**) MOG^35–55^-specific 2D2 CD4+ T cells or (**e**) WT or Cat Z^−/−^ MOG^35–55^-specific 2D2 CD4+ T cells. Activation of WT and Cat Z^−/−^ MOG^35–55^-specific 2D2 CD4+ T cells was assessed via CD4+ T cell CD69 surface expression after 16 h co-incubation with APCs (*n* = 3–6). Data presented as mean+/− SEM; no significant differences (unpaired Student’s *t* test, *p* > 0.05) from WT controls were observed

**Fig. 4:**
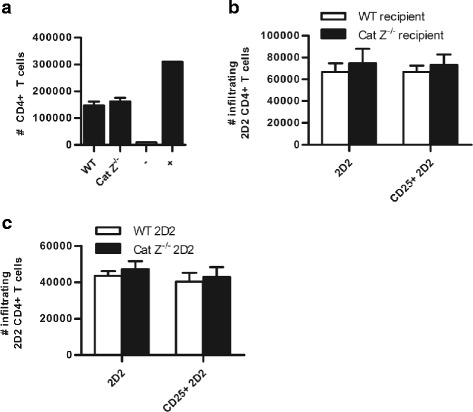
CD4+ T cells deficient in cathepsin Z exhibit proficient migration in vitro; and efficient trafficking, infiltration, and reactivation in the CNS. **a** To determine whether deficiency of cathepsin Z affected the ability of CD4+ T cells to undergo chemotaxis in response to CXCL9, CD4+ T cells were isolated from the spleens of WT and Cat Z^−/−^ mice and given 1 h to migrate through an ICAM coated Transwell plate in response to CXCL9 (*n* = 4). The negative control (−) well had no CXCL9. The positive control (+) well had no filter, allowing all CD4+ T cells to migrate through to the bottom of the Transwell. **b** To evaluate the ability of CD4+ T cells to infiltrate the CNS in WT and Cat Z^−/−^ mice, MOG^35–55^-specific 2D2 CD4+ T cells were isolated and expanded ex vivo using IL-12 and MOG^35–55^ for 48 h before adoptive transfer into WT and Cat Z^−/−^ recipient mice; alternatively, (**c**) to examine the capacity of Cat Z^−/−^ CD4+ T cells to infiltrate the CNS of WT mice, WT and Cat Z^−/−^ MOG^35–55^-specific 2D2 CD4+ T cells were isolated, expanded and adoptively transferred into WT recipients. **b**-**c** Six days following adoptive transfer, the 2D2 CD4+ T cells were isolated from the CNS using a discontinuous Percoll gradient, identified by flow cytometry (CD4+, Vα3.2+) and evaluated for the expression of the activation marker CD25 (*n* = 4). Data presented as mean+/− SEM; no significant differences (unpaired Student’s *t* test, *p* > 0.05) from the WT control were observed

To determine whether cathepsin Z-deficiency impacts EAE through perturbation of the processing and/or presentation of the autoantigen MOG, APCs derived from WT and Cat Z^−/−^ mice were incubated with pre-processed MOG peptide (MOG^35–55^) or unprocessed MOG protein (MOG^1–125^) and co-cultured with MOG^35–55^-specific CD4+ T cells. Both BMMØs and BMDCs from Cat Z^−/−^ mice were able to activate and mature in response to proinflammatory stimuli and could efficiently activate MOG^35–55^-specific CD4+ T cells in an antigen-specific fashion (as determined by expression of the early T cell activation marker, CD69) (Fig. 3a–d). Consistent with an insignificant role for cathepsin Z in phagosomal proteolysis, these data demonstrate that cathepsin Z does not impact the processing or presentation of the autoantigen MOG, suggesting that its role in the pathogenesis of murine EAE is mediated through a mechanism not previously attributed to lysosomal cysteine cathepsins [39]. To investigate a potential role of cathepsin Z in T cell functions germane to EAE, Cat Z^−/−^ CD4+ T cells were evaluated for their ability to activate, chemotax and respond to MOG. It was found that CD4+ T cells isolated from WT and Cat Z^−/−^ mice were equally able to activate in a MOG^35–55^-specific fashion (Fig. 3e). Similarly, the absence of cathepsin Z did not affect the ability of CD4+ T cells to chemotax in response to CXCL9 (Fig. 4a). Consistent with these findings, adoptively transferred WT and Cat Z^−/−^ MOG^35–55^-specific CD4+ T cells traversed the blood-brain-barrier and responded to the presence of endogenous MOG within the CNS in comparable fashions (Fig. 4b, c).

### Mice deficient in cathepsin Z are unable to efficiently generate IL-1β during EAE and in response to inflammasome activation, and show deficiencies in Th17 polarization

**Fig. 5:**
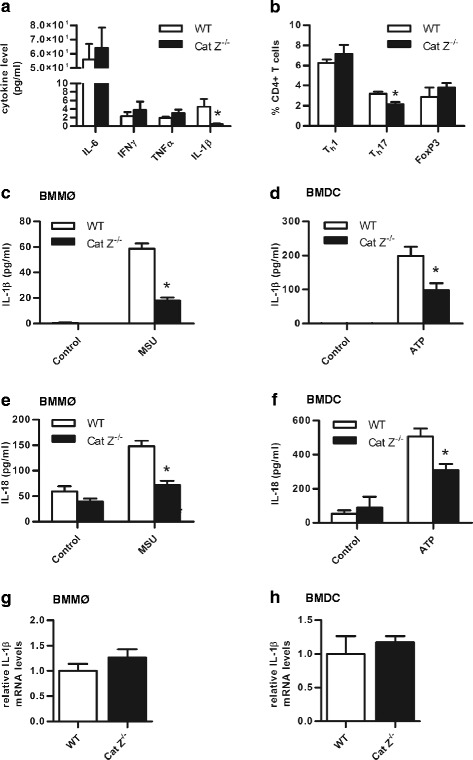
Mice deficient in cathepsin Z have dramatically reduced circulating IL-1β during EAE and attenuated Th17 responses in vivo; consistently, APCs deficient in cathepsin Z are unable to efficiently generate IL-1β and IL-18 in vitro. **a** Proinflammatory cytokine levels in WT and Cat Z^−/−^ EAE serum 15 days post induction (*n* = 3–6). **b** The percentage of Th1 (IFNγ+), Th17 (IL-17+), and FoxP3+ CD4+ T cells isolated from the inguinal lymph nodes of WT and Cat Z^−/−^ mice 6 days after injection with CFA (*n* = 3). **c**, **d** Concentration of IL-1β within the supernatant of WT and Cat Z^−/−^ (**c**) BMMØ, and (**d**) BMDC after priming with LPS and subsequent exposure to NLRP3-inflammasome activators (**c**) monosodium urate (MSU) or (**d**) ATP as quantified by ELISA (*n* = 5). **e**, **f** Concentration of IL-18 within the supernatant of WT and Cat Z^−/−^ (**e**) BMMØ, and (**f**) BMDC after priming with LPS and subsequent exposure to the NLRP3-inflammasome activators (**e**) MSU or (**f**) ATP as quantified by ELISA (*n* = 5). **g**, **h** IL-1β mRNA levels of WT and Cat Z^−/−^ (**g**) BMMØ and (**h**) BMDCs stimulated with LPS as determined by qPCR (*n* = 3–5). Data presented as mean +/− SEM; significant differences (unpaired Student’s *t* test, *p* < 0.05) from the WT control are denoted by *asterisks*

Although it is expected that circulating levels of proinflammatory cytokines would correspond to a decrease in neuroinflammation during EAE, it was noted that Cat Z^−/−^ mice had dramatically lower IL-1β but comparable levels of other circulating proinflammatory cytokines during EAE (Fig. 5a). Since IL-1β has been shown to be critically important in the development of autoreactive Th17 cells implicated in the pathogenesis of MS and EAE [40–46], we set out to determine whether mice deficient in cathepsin Z could efficiently generate a Th17 response. When cathepsin Z-deficient mice were challenged with CFA in the absence of antigen, the draining lymph nodes contained equivalent proportions of Th1 and FoxP3+ CD4+ T cells, but proportionately reduced numbers of Th17 cells (Fig. 5b). Direct examination of a role for cathepsin Z in IL-1β production by APCs revealed significant reductions in secreted IL-1β and IL-18 following the induction of the NLRP3 inflammasome, despite equivalent expression of IL-1β mRNA in response to LPS (Fig. 5c–h). These data are consistent with a non-redundant role for cathepsin Z in the processing of IL-1β, and the IL-1β-dependent Th17 response accountable for enhanced neuroinflammation during EAE [40–47].

## Discussion

This study provides the first experimental evidence that cathepsin Z positively contributes to neuroinflammation and the pathogenesis of EAE in a non-redundant fashion. Although phagocytic APCs express cathepsin Z at high levels, we established that cathepsin Z’s role in EAE is not mediated through antigen processing and presentation. Consistently, we showed that the enzyme’s contribution to overall proteolysis within phagolysosomes of APCs is minimal to absent—demonstrating that general protein turnover is not a primary function of this carboxypeptidase. Interestingly, we showed that cathepsin Z-deficiency leads to reduced serum IL-1β and Th17 polarization during EAE, and lowered IL-1β production by APCs in response to NLRP3-activating stimulus.

Over the past decade, several pieces of evidence have emerged that implicate cathepsin Z in neuroinflammation. Cathepsin Z has been shown to be disproportionately expressed and secreted by both microglia and astrocytes in response to neuronal damage and inflammatory stimulus, both in culture and in vivo [10–13]. It was reported that dendritic cells in the aging brains of mice had increased expression of cathepsin Z that correlated with known markers of neuroinflammation [48]. Furthermore, a comprehensive comparative gene expression analysis of mouse models of MS (EAE), Alzheimer’s disease and stroke, found that cathepsin Z is one of eighteen genes whose expression is increased in all three models of neuroinflammation [38]. Whilst the expression and release of cathepsin Z has been shown to be associated with neuroinflammation, and more recently, epigenetic dysregulation of cathepsin Z with multiple sclerosis, this study provides the first experimental evidence that cathepsin Z positively contributes to neuroinflammation. Moreover, unlike typical lysosomal cysteine cathepsins, we show that cathepsin Z’s role in EAE is non-redundant, and is not mediated through antigen processing and presentation, but likely through perturbation of the IL-1β—Th17 pathway.

Beyond the CNS, cathepsin Z has been associated with inflammatory conditions in other tissues. Levels of cathepsin Z (and procathepsin Z) have been shown to be significantly increased in the plasma and serum of patients who had suffered multiple traumas—which correlated with severity—and have been proposed to be used as a clinical marker for systemic inflammation [49]. Cathepsin Z has been shown to be upregulated in human gastric mucosa that is chronically infected with *Helicobacter pylori*, and to contribute to chronic inflammation and the development of gastric metaplasia in a mouse model of *Helicobacter*-induced gastritis [50, 51]. These studies suggest a broader role for cathepsin Z in inflammation, potentially mediated through a common pathway involving the generation of IL-1β.

While this study identifies a mechanistic role for cathepsin Z in neuroinflammation and MS, several key questions remain unanswered. How does cathepsin Z, a strict carboxypeptidase, promote IL-1β generation, and where does cathepsin Z physically act in this pathway? In 2008, Hornung et al. reported that phagosomal destabilization and cytosolic activity of cathepsin B (or L) act to trigger inflammasome assembly in response to silica and alum, mainly based on the use of cysteine cathepsin inhibitors [52]. Subsequently, others have shown that APCs deficient in cathepsin B or L show unaltered levels of IL-1β following inflammasome activation in response to a variety of crystalline and soluble stimuli [33, 47], and that the involvement of these “typical” lysosomal cysteine cathepsins in inflammasome activation or efficiency is likely redundant. The non-redundant role of cathepsin Z in IL-1β generation suggests that this cysteine protease acts to promote IL-1β generation through a mechanism distinct from that used by cathepsin B and L within the cytosol. Alternatively, it is possible that secreted cathepsin Z (or pro-cathepsin Z) acts to enhance IL-1β generation through extracellular pathways, as has been demonstrated for cathepsin C [53]. However, since cathepsin Z is a strict carboxypeptidase and cathepsin Z-deficient APCs show reduced IL-1β release in in vitro conditions, it is unlikely that cathepsin Z directly processes extracellular IL-1β in a caspase 1-independent fashion. Another intriguing possibility is that procathepsin Z, or the cleaved proregion of cathepsin Z, enhances or triggers the assembly of the NLRP3 inflammasome through the activation of integrins on the surface of APCs in an autocrine or paracrine fashion through its evolutionarily-conserved RGD integrin binding domain [9]. Indeed, the surface protein Td92 of the periodontopathogen *Treponema denticola*, as well as the RGD-containing cysteine proteinase 5 of *Entamoeba histolytica*, have been shown to enhance inflammasome-mediated IL-1β generation through RGD-dependent activation of the α5β1 integrin on macrophages [54–56].

Although further insight into how cathepsin Z enhances IL-1β generation during neuroinflammation and what leads to the hypomethylation of the CTSZ locus in the human brain is warranted, the data generated in this study directly implicate cathepsin Z in the promotion of IL-1β-driven neuroinflammation. Moreover, these findings provide experimental evidence to support the proposal that epigenetic dysregulation of cathepsin Z within the human brain may increase an individual’s susceptibility to MS [2].

## Additional file

Additional file 1: Figure S1. Mice deficient in cathepsin Z exhibit clinical signs of EAE compared to WT siblings. Although the Cat Z^-/-^ mice used in this study were fully backcrossed to C57BL/6 (WT), to definitively rule out any anomalies resulting from background genetics or environment, EAE was induced with 50 μg MOG^35-55^ in CFA and 300 ng Pertussis Toxin (day 0 and 2) and scored on a standard 5 point scale. (*n* = 6–9). Data presented as mean+/- SEM; significant differences (Mann–Whitney *U﻿* test, *p* < 0.05) from the WT control are denoted by asterisks (*). (PNG 140 kb)

## Abbreviations

2D2: C57BL/6-Tg(Tcra2D2,Tcrb2D2)1Kuch/J
2-ME: β-mercaptoethanol
APCs: Antigen presenting cells
BMDCs: Bone marrow-derived dendritic cells
BMMØs: Bone marrow-derived macrophages
Cat Z^−/−^: Cathepsin Z-deficient mice
CFA: Complete Freund’s adjuvant
CNS: Central nervous system
CTSZ: Cathepsin Z
EAE: Experimental autoimmune encephalomyelitis
FSC/SSC: Forward scatter/side scatter
HE: Hematoxylin and eosin
LFB: Luxol fast blue
LNC: Lymph node cells
MS: Multiple sclerosis
N2A: Neuro-2a
pMØs: Peritoneal macrophages
qPCR: Quantitative PCR
rIFNγ: Recombinant IFNγ
WT: Wildtype

## References

[1] Consortium IMSG, 2 WTCCC (2011). Genetic risk and a primary role for cell-mediated immune mechanisms in multiple sclerosis. Nature.

[2] Huynh JL, Garg P, Thin TH, Yoo S, Dutta R, Trapp BD, Haroutunian V, Zhu J, Donovan MJ, Sharp AJ (2014). Epigenome-wide differences in pathology-free regions of multiple sclerosis-affected brains. Nat Neurosci.

[3] Nägler DK, Ménard R (1998). Human cathepsin X: a novel cysteine protease of the papain family with a very short proregion and unique insertions. FEBS Lett.

[4] Santamaria I, Velasco G, Pendas AM, Fueyo A, Lopez-Otin C (1998). Cathepsin Z, a novel human cysteine proteinase with a short propeptide domain and a unique chromosomal location. J Biol Chem.

[5] Nägler DK, Zhang R, Tam W, Sulea T, Purisima EO, Ménard R (1999). Human cathepsin X: A cysteine protease with unique carboxypeptidase activity. Biochemistry.

[6] Cooke GS, Campbell SJ, Bennett S, Lienhardt C, McAdam KP, Sirugo G, Sow O, Gustafson P, Mwangulu F, van Helden P (2008). Mapping of a novel susceptibility locus suggests a role for MC3R and CTSZ in human tuberculosis. Am J Respir Crit Care Med.

[7] Lechner AM, Assfalg-Machleidt I, Zahler S, Stoeckelhuber M, Machleidt W, Jochum M, Nägler DK (2006). RGD-dependent binding of procathepsin X to integrin αvβ3 mediates cell-adhesive properties. J Biol Chem.

[8] Xiao T, Takagi J, Coller BS, Wang J-H, Springer TA (2004). Structural basis for allostery in integrins and binding to fibrinogen-mimetic therapeutics. Nature.

[9] Akkari L, Gocheva V, Kester JC, Hunter KE, Quick ML, Sevenich L, Wang HW, Peters C, Tang LH, Klimstra DS (2014). Distinct functions of macrophage-derived and cancer cell-derived cathepsin Z combine to promote tumor malignancy via interactions with the extracellular matrix. Genes Dev.

[10] Hwang S-Y, Yoo B-C, Jung J-w, Oh E-S, Hwang J-S, Shin J-A, Kim S-Y, Cha S-H, Han I-O (2009). Induction of glioma apoptosis by microglia-secreted molecules: The role of nitric oxide and cathepsin B. Biochim Biophys Acta.

[11] Greco TM, Seeholzer SH, Mak A, Spruce L, Ischiropoulos H (2010). Quantitative mass spectrometry-based proteomics reveals the dynamic range of primary mouse astrocyte protein secretion. J Proteome Res.

[12] Wendt W, Schulten R, Stichel CC, Lübbert H (2009). Intra‐versus extracellular effects of microglia‐derived cysteine proteases in a conditioned medium transfer model. J Neurochem.

[13] Glanzer JG, Enose Y, Wang T, Kadiu I, Gong N, Rozek W, Liu J, Schlautman JD, Ciborowski PS, Thomas MP (2007). Genomic and proteomic microglial profiling: pathways for neuroprotective inflammatory responses following nerve fragment clearance and activation. J Neurochem.

[14] Bettelli E, Pagany M, Weiner HL, Linington C, Sobel RA, Kuchroo VK (2003). Myelin oligodendrocyte glycoprotein-specific T cell receptor transgenic mice develop spontaneous autoimmune optic neuritis. J Exp Med.

[15] Sevenich L, Schurigt U, Sachse K, Gajda M, Werner F, Muller S, Vasiljeva O, Schwinde A, Klemm N, Deussing J (2010). Synergistic antitumor effects of combined cathepsin B and cathepsin Z deficiencies on breast cancer progression and metastasis in mice. Proc Natl Acad Sci U S A.

[16] Allan ER, Tailor P, Balce DR, Pirzadeh P, McKenna NT, Renaux B, Warren AL, Jirik FR, Yates RM (2014). NADPH oxidase modifies patterns of MHC class II-restricted epitopic repertoires through redox control of antigen processing. J Immunol.

[17] Stromnes IM, Goverman JM (2006). Active induction of experimental allergic encephalomyelitis. Nat Protoc.

[18] Yates RM, Hermetter A, Russell DG (2005). The kinetics of phagosome maturation as a function of phagosome/lysosome fusion and acquisition of hydrolytic activity. Traffic.

[19] Balce DR, Li B, Allan ER, Rybicka JM, Krohn RM, Yates RM (2011). Alternative activation of macrophages by IL-4 enhances the proteolytic capacity of their phagosomes through synergistic mechanisms. Blood.

[20] Savina A, Jancic C, Hugues S, Guermonprez P, Vargas P, Moura IC, Lennon-Dumenil AM, Seabra MC, Raposo G, Amigorena S (2006). NOX2 controls phagosomal pH to regulate antigen processing during crosspresentation by dendritic cells. Cell.

[21] Rybicka JM, Balce DR, Chaudhuri S, Allan ER, Yates RM (2012). Phagosomal proteolysis in dendritic cells is modulated by NADPH oxidase in a pH-independent manner. Embo J.

[22] Karasuyama H, Melchers F (1988). Establishment of mouse cell lines which constitutively secrete large quantities of interleukin 2, 3, 4 or 5, using modified cDNA expression vectors. Eur J Immunol.

[23] Pineda-Torra I, Gage M, de Juan A, Pello OM (2015). Isolation, Culture, and Polarization of Murine Bone Marrow-Derived and Peritoneal Macrophages. Methods Mol Biol.

[24] Shen Z, Reznikoff G, Dranoff G, Rock KL (1997). Cloned dendritic cells can present exogenous antigens on both MHC class I and class II molecules. J Immunol.

[25] Mantegazza AR, Guttentag SH, El-Benna J, Sasai M, Iwasaki A, Shen H, Laufer TM, Marks MS (2012). Adaptor protein-3 in dendritic cells facilitates phagosomal toll-like receptor signaling and antigen presentation to CD4(+) T cells. Immunity.

[26] Rosenthal KM, Edwards LJ, Sabatino JJ, Hood JD, Wasserman HA, Zhu C, Evavold BD (2012). Low 2- dimensional CD4 T cell receptor affinity for myelin sets in motion delayed response kinetics. PLoS One.

[27] Atif SM, Uematsu S, Akira S, McSorley SJ (2014). CD103-CD11b + dendritic cells regulate the sensitivity of CD4 T-cell responses to bacterial flagellin. Mucosal Immunol.

[28] Charo IF, Ransohoff RM (2006). The many roles of chemokines and chemokine receptors in inflammation. N Engl J Med.

[29] Williams JL, Kithcart AP, Smith KM, Shawler T, Cox GM, Whitacre CC (2011). Memory cells specific for myelin oligodendrocyte glycoprotein (MOG) govern the transfer of experimental autoimmune encephalomyelitis. J Neuroimmunol.

[30] Agrawal SM, Silva C, Tourtellotte WW, Yong VW (2011). EMMPRIN: a novel regulator of leukocyte transmigration into the CNS in multiple sclerosis and experimental autoimmune encephalomyelitis. J Neurosci.

[31] Bernhardt A, Kuester D, Roessner A, Reinheckel T, Krueger S (2010). Cathepsin X-deficient gastric epithelial cells in co-culture with macrophages: characterization of cytokine response and migration capability after Helicobacter pylori infection. J Biol Chem.

[32] Levesque SA, Kukulski F, Enjyoji K, Robson SC, Sevigny J (2010). NTPDase1 governs P2X7-dependent functions in murine macrophages. Eur J Immunol.

[33] Hari A, Zhang Y, Tu Z, Detampel P, Stenner M, Ganguly A, Shi Y (2014). Activation of NLRP3 inflammasome by crystalline structures via cell surface contact. Sci Rep.

[34] Brand‐Schieber E, Werner P, Iacobas DA, Iacobas S, Beelitz M, Lowery SL, Spray DC, Scemes E (2005). Connexin43, the major gap junction protein of astrocytes, is down‐regulated in inflamed white matter in an animal model of multiple sclerosis. J Neurosci Res.

[35] Rudensky A, Beers C. Lysosomal cysteine proteases and antigen presentation. Ernst Schering Res Found Workshop. 2006;56:81–95. 10.1007/3-540-37673-9_5 16329647

[36] Honey K, Rudensky AY (2003). Lysosomal cysteine proteases regulate antigen presentation. Nat Rev Immunol.

[37] Hsing LC, Rudensky AY (2005). The lysosomal cysteine proteases in MHC class II antigen presentation. Immunol Rev.

[38] Tseveleki V, Rubio R, Vamvakas S-S, White J, Taoufik E, Petit E, Quackenbush J, Probert L (2010). Comparative gene expression analysis in mouse models for multiple sclerosis, Alzheimer’s disease and stroke for identifying commonly regulated and disease-specific gene changes. Genomics.

[39] Allan ER, Yates RM (2015). Redundancy between Cysteine Cathepsins in Murine Experimental Autoimmune Encephalomyelitis. PLoS One.

[40] Lalor SJ, Dungan LS, Sutton CE, Basdeo SA, Fletcher JM, Mills KH (2011). Caspase-1–processed cytokines IL-1β and IL-18 promote IL-17 production by γδ and CD4 T cells that mediate autoimmunity. J Immunol.

[41] Shaw PJ, Lukens JR, Burns S, Chi H, McGargill MA, Kanneganti T-D (2010). Cutting edge: critical role for PYCARD/ASC in the development of experimental autoimmune encephalomyelitis. J Immunol.

[42] Shaw PJ, McDermott MF, Kanneganti T-D (2011). Inflammasomes and autoimmunity. Trends Mol Med.

[43] Gris D, Ye Z, Iocca HA, Wen H, Craven RR, Gris P, Huang M, Schneider M, Miller SD, Ting JP-Y (2010). NLRP3 plays a critical role in the development of experimental autoimmune encephalomyelitis by mediating Th1 and Th17 responses. J Immunol.

[44] Ikeda S, Saijo S, Murayama MA, Shimizu K, Akitsu A, Iwakura Y (2014). Excess IL-1 Signaling Enhances the Development of Th17 Cells by Downregulating TGF-β–Induced Foxp3 Expression. J Immunol.

[45] Lovett-Racke AE, Yang Y, Racke MK (2011). Th1 versus Th17: are T cell cytokines relevant in multiple sclerosis?. Biochim Biophys Acta.

[46] Murphy ÁC, Lalor SJ, Lynch MA, Mills KH (2010). Infiltration of Th1 and Th17 cells and activation of microglia in the CNS during the course of experimental autoimmune encephalomyelitis. Brain Behav Immun.

[47] Orlowski GM, Colbert JD, Sharma S, Bogyo M, Robertson SA, Rock KL (2015). Multiple Cathepsins Promote Pro-IL-1beta Synthesis and NLRP3-Mediated IL-1beta Activation. J Immunol.

[48] Stichel CC, Luebbert H (2007). Inflammatory processes in the aging mouse brain: participation of dendritic cells and T-cells. Neurobiol Aging.

[49] Nägler DK, Lechner AM, Oettl A, Kozaczynska K, Scheuber H-P, Gippner-Steppert C, Bogner V, Biberthaler P, Jochum M (2006). An enzyme-linked immunosorbent assay for human cathepsin X, a potential new inflammatory marker. J Immunol Methods.

[50] Krueger S, Kalinski T, Hundertmark T, Wex T, Küster D, Peitz U, Ebert M, Nägler DK, Kellner U, Malfertheiner P (2005). Up‐regulation of cathepsin X in Helicobacter pylori gastritis and gastric cancer. J Pathol.

[51] Krueger S, Bernhardt A, Kalinski T, Baldensperger M, Zeh M, Teller A, Adolf D, Reinheckel T, Roessner A, Kuester D (2013). Induction of premalignant host responses by cathepsin x/z-deficiency in Helicobacter pylori-infected mice. PLoS One.

[52] Hornung V, Bauernfeind F, Halle A, Samstad EO, Kono H, Rock KL, Fitzgerald KA, Latz E (2008). Silica crystals and aluminum salts activate the NALP3 inflammasome through phagosomal destabilization. Nat Immunol.

[53] Kono H, Orlowski GM, Patel Z, Rock KL (2012). The IL-1-dependent sterile inflammatory response has a substantial caspase-1-independent component that requires cathepsin C. J Immunol.

[54] Mortimer L, Moreau F, Cornick S, Chadee K (2015). The NLRP3 Inflammasome Is a Pathogen Sensor for Invasive Entamoeba histolytica via Activation of alpha5beta1 Integrin at the Macrophage-Amebae Intercellular Junction. PLoS Pathog.

[55] Hou Y, Mortimer L, Chadee K (2010). Entamoeba histolytica cysteine proteinase 5 binds integrin on colonic cells and stimulates NFkappaB-mediated pro-inflammatory responses. J Biol Chem.

[56] Jun HK, Lee SH, Lee HR, Choi BK (2012). Integrin alpha5beta1 activates the NLRP3 inflammasome by direct interaction with a bacterial surface protein. Immunity.

