# Supplementary figures and images for: A role for cathepsin Z in neuroinflammation provides mechanistic support for an epigenetic risk factor in multiple sclerosis
Source: J Neuroinflammation. 2017 May 10;14:103. doi: 10.1186/s12974-017-0874-x (PMC5424360; doi:10.1186/s12974-017-0874-x)

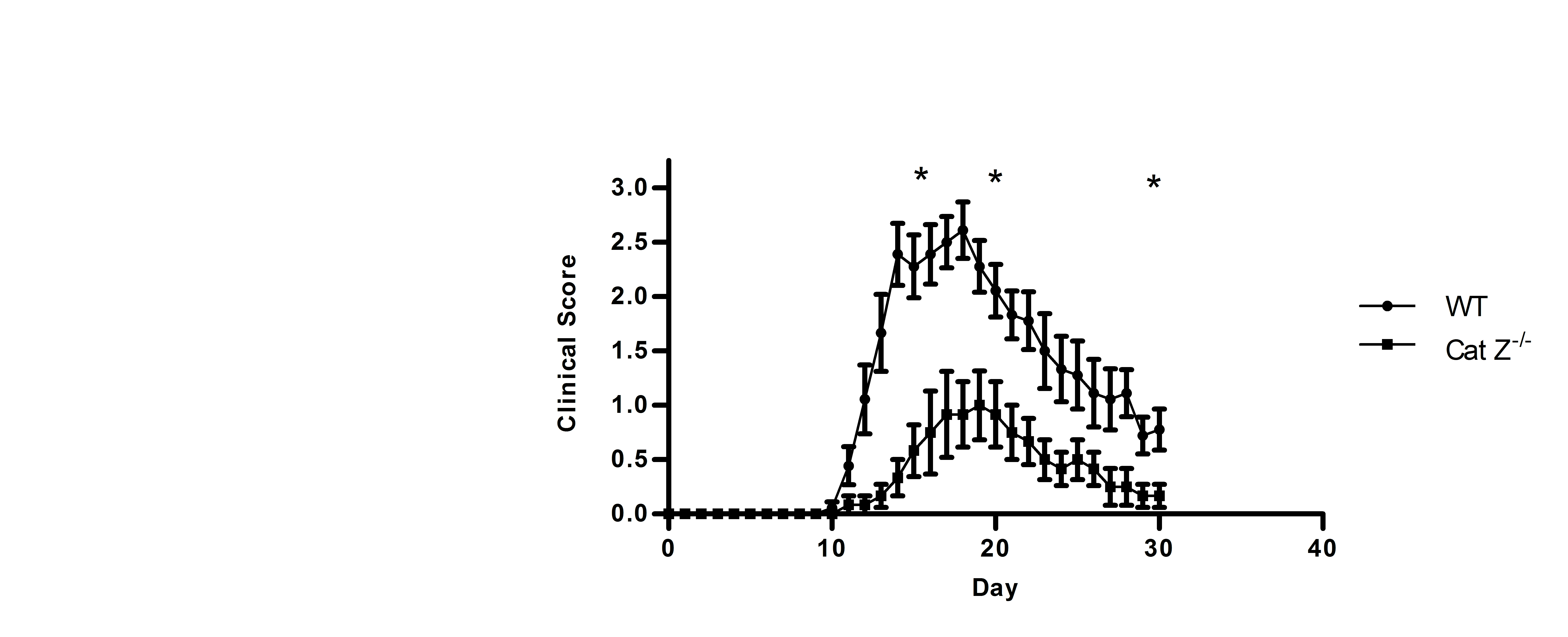

Supplement: Additional file 1: Figure S1. — Mice deficient in cathepsin Z exhibit clinical signs of EAE compared to WT siblings. Although the Cat Z-/- mice used in this study were fully backcrossed to C57BL/6 (WT), to definitively rule out any anomalies resulting from background genetics or environment, EAE was induced with 50 μg MOG35-55 in CFA and 300 ng Pertussis Toxin (day 0 and 2) and scored on a standard 5 point scale. (n = 6–9). Data presented as mean+/- SEM; significant differences (Mann–Whitney U﻿ test, p < 0.05) from the WT control are denoted by asterisks (*). (PNG 140 kb) [file 12974_2017_874_MOESM1_ESM.png]
